# The Cologne ECMM Excellence Center: A Two-Year Analysis of External Consultation Service for Invasive Fungal Infections

**DOI:** 10.1007/s11046-023-00822-1

**Published:** 2024-03-11

**Authors:** Jon Salmanton-García, Philipp Koehler, Jan-Hendrik Grothe, Sibylle C. Mellinghoff, Ertan Sal, Michaela Simon, Jannik Stemler, Oliver A. Cornely, Rosanne Sprute

**Affiliations:** 1 Faculty of Medicine and University Hospital Cologne, Institute of Translational Research, Cologne Excellence Cluster on Cellular Stress Responses in Aging-Associated Diseases, University of Cologne, Herderstraße 52, 50931 Cologne, Germany; 2 Department I of Internal Medicine, Faculty of Medicine and University Hospital Cologne, Excellence Center for Medical Mycology, University of Cologne, Cologne, Germany; 3 German Centre for Infection Research (DZIF), Partner Site Bonn-Cologne, Cologne, Germany; 4 Faculty of Medicine and University Hospital Cologne, Clinical Trials Centre Cologne (ZKS Köln), University of Cologne, Cologne, Germany

**Keywords:** Medical mycology, Clinical management, Consultation, Antifungal diagnosis, Antifungal treatment

## Abstract

The European Confederation of Medical Mycology (ECMM), formed due to the surge in invasive fungal infections (IFI), initiated the Excellence Centers program in 2016 to guide stakeholders to leading medical mycology sites. This report focuses on the Cologne ECMM Excellence Center, recognized with Diamond status for active global involvement in 2017. The center offers free consultation via email and phone, responding within 24 h for life-threatening IFI, collecting data on origin, pathogens, infection details, and more. Over two years, 189 requests were received globally, predominantly from Germany (85%), mainly involving *Aspergillus* spp., Mucorales, and *Candida* spp. Fungal mixed infections occurred in 4% of cases. The center's service effectively addresses IFI challenges, advocating for a comprehensive study encompassing all ECMM Excellence Centers to enhance global mycological care. Proactive expansion of consultancy platforms is crucial, with future analyses needed to assess expert advice's impact on patient outcomes.

## Introduction

The field of medical mycology has become increasingly vital in response to the rising prevalence of invasive fungal infections (IFI) and their impact on global health [1]. To give these ambitions a platform, the European Confederation of Medical Mycology (ECMM) was established in 1993, [2] as an umbrella organization for mycological societies Europewide, focused both on the laboratory and clinical perspectives. Recognizing the significance of maintaining high-quality standards and promoting excellence in clinical and microbiological mycology, the ECMM introduced the ECMM Excellence Centers initiative in 2016. [3]

The ECMM Excellence Centers initiative aims to certify institutions for outstanding achievements in medical mycology. [4, 5] The program encompasses four levels of recognition: Blue, Silver, Gold, and the Diamond status, while the Diamond status can only be obtained when beyond clinical and microbiological excellence, a center actively contributes to ECMM studies [4, 6–19]. These designations are awarded based on a rigorous evaluation process conducted by a designated committee of the ECMM. Centers aspiring to attain excellence status can apply for evaluation, and upon successful review and on-site audit, they receive esteemed recognition within the medical mycology community (https://www.ecmm.info/ecmm-excellence-centers/) [20]. As of August 2023, 13 centers in 9 countries have achieved ECMM Excellence Center status, with 12 at Diamond and one at Silver. Four sites are under evaluation [3]. In 2017, the University of Cologne achieved Diamond status, exemplifying excellence in medical mycology, including launching an expert consultation service on invasive fungal infections (https://www.ecmm.info/ecmm-excellence-centers/) [3].

This manuscript summarizes the Cologne ECMM Excellence Center's consultation service experiences and outcomes from August 2021 to July 2023. By sharing these insights, the center aims to provide valuable knowledge on establishing and managing such services, emphasizing the importance of expert consultation in making evidence-based decisions for patients with IFI.

## Methods

The Cologne ECMM Excellence Center earned the Diamond status by actively participating in various initiatives. These included coordinating multicenter international ECMM projects [4, 8–17, 19, 21], developing the EQUAL Score Cards [9, 11, 12, 16–18, 22], managing the Fungiscope®—Global Emerging Fungal Infection Registry [23–25], and engaging in One World—One Guideline development for IFI management [7, 8, 13, 19, 26, 27]. The center also established a free-of-charge global consultation system to provide expert advice and rapid feedback to healthcare professionals dealing with IFI. Furthermore, there is an internal scheduled weekly meeting to delve into relevant aspects of patients contemporaneously under study. This meeting includes a diverse team of professionals as required by the characteristics of the patient under discussion, including epidemiologists, hematologists, microbiologists, mycologists, oncologists, or radiologists. This composition enables a thorough and comprehensive discussion.

Through the global consultation platform, physicians from all around the world can access the Cologne ECMM Excellence Center. This platform provides them with the chance to seek guidance by reaching out to the center via email (ecmm-ec@uk-koeln.de) or phone (+ 49 221 478 85,523) in instances of suspected or confirmed IFI cases in their care. The primary principle of the center is to ensure a response to all inquiries within 24 h for IFI that pose a life-threatening risk, thus providing fast and reliable assistance for the management of complex scenarios, as timely intervention holds the potential to greatly impact patient outcomes.

During initial data collection, we gather key information about the case, including patient clinical details, radiological findings, microbiological evidence, prior or ongoing IFI treatments, underlying medical conditions, and any allergies. Our imaging procedures utilize a secure cloud-based system (easy Radiology AG, Cologne, Germany) for detailed image collection, ensuring patient data confidentiality. After anonymizing and encrypting images with a unique key, the key is sent via email to the uploader, and access requires explicit permission. The provided advice may encompass diagnostics, laboratory services, therapy options, drug level measurements, follow-up assessments, and potential patient participation in clinical trials.

## Results

**Fig. 1:**
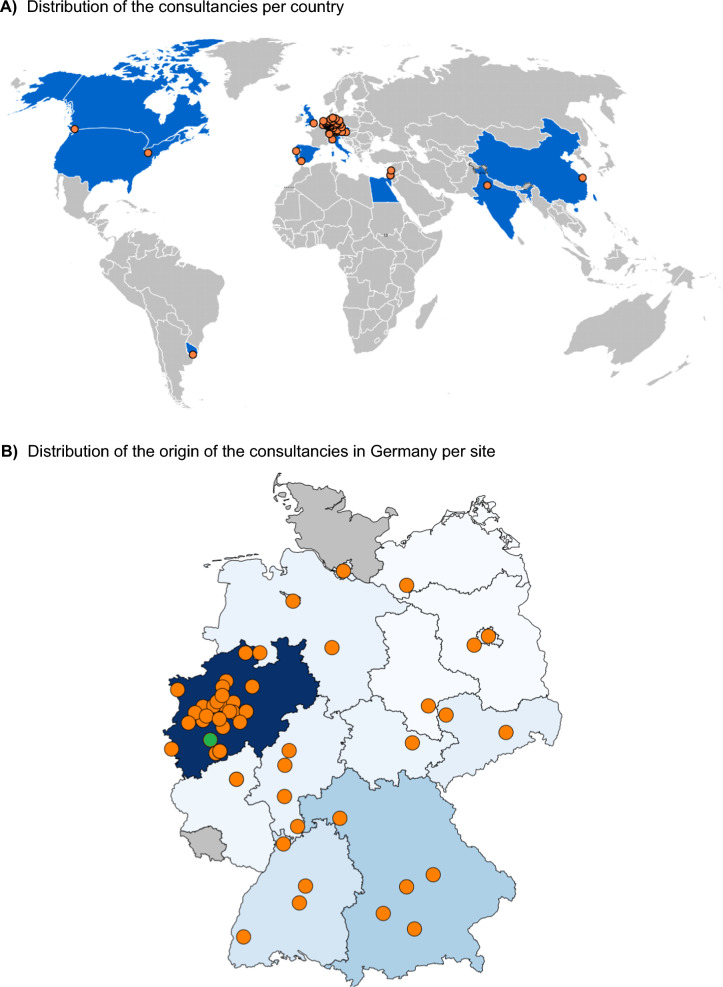

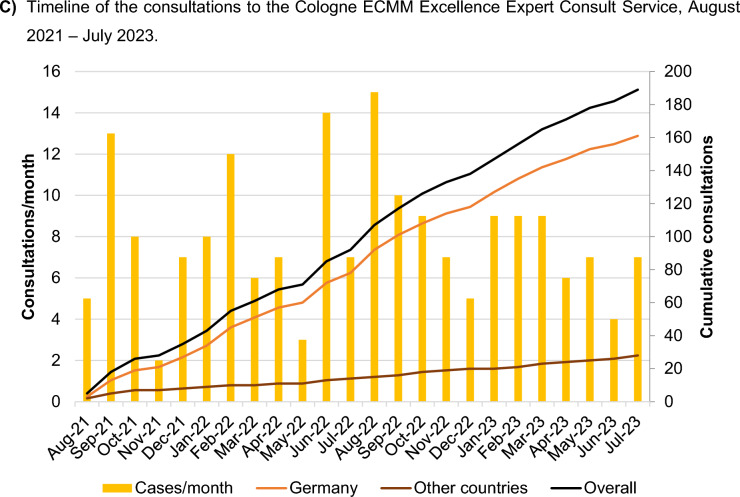
Distribution of the consultancies per origin and month since Cologne ECMM Excellence Center activation. **a** Distribution of the consultancies per country. Number of consultancies per country: Germany (n = 162), Israel (n = 6), Austria (n = 3), Belgium, India, and Portugal (n = 2, each), and Canada, China, Czech Republic, Egypt, Italy, Lebanon, Spain, Switzerland, United Kingdom, United States, and Uruguay (n = 1, each). **b** Distribution of the origin of the consultancies in Germany per site. Blue corresponds to Federal States where consultations have originated. The intensity of the blue shade indicates the number of consultations from each state, with darker blue signifying a higher number of consultations. Grey color denotes states with no consultations recorded. Additionally, a green dot marks the location of the Cologne ECMM Excellence Centre, while orange dots indicate the origin sites of consultancy inquiry. Consultations have come from North Rhine-Westphalia (n = 75), Bavaria (n = 25), Baden-Württemberg (n = 14), Saxony (n = 8), Hamburg, Hesse, and Lower Saxony (n = 6, each), Berlin and Thuringia (n = 5, each), Rhineland-Palatinate (n = 4), and Brandenburg, Bremen, Mecklenburg-Western Pomerania, and Saxony-Anhalt (n = 1, each). No consultations from Saarland or Schleswig–Holstein. Timeline of the consultations to the Cologne ECMM Excellence Expert Consult Service, August 2021–July 2023

In two years, the Cologne ECMM Excellence Center responded to 189 consultations from 17 countries around the world (Fig. 1A). The majority (85.2%) came from Germany (Fig. 1B). Afterwards, from other European countries and rest of the world (n = 14, each 7.4%). Of these 189 global consultations, 63.0% were related to IFI, primarily involving *Aspergillus* spp. (31.2%), Mucorales (18.0%), and *Candida* spp. (13.8%). Fungal coinfections occurred in 4.3% of cases, with *Aspergillus* spp. prevalent in 71.4%. Some cases (1.9%) involved concurrent fungal, bacterial, or viral infections. Additionally, 18 radiological pictures from six patients with complex conditions, mainly in Germany, were comprehensively analyzed.

A detailed analysis of consultations from German sites revealed that 40.7% (77/161) were within 100 km of the Cologne ECMM Excellence Center. Consultations came from 14 of the 16 Federal States, with North Rhine-Westphalia (46.6%), Bavaria (15.5%), and Baden-Württemberg (8.7%) contributing the most. At the pathogen level, consultations from North Rhine-Westphalia were primarily for *Aspergillus* spp. (55.8%), Mucorales (66.7%), *Candida* spp. (66.7%), and *Fusarium* spp. (41.7%). Bavaria had a higher frequency of consultations related to *Lomentospora*/*Scedosporium* spp. infections (55.6%).

The ECMM expert consult service was also used to enable enrolment in clinical trials with novel treatment options [28] for patients with refractory or difficult-to-treat IFI which was successful in four cases, leading to a referral of these patients to the University Hospital Cologne.

The temporal distribution of consultations showed fluctuations in demand over the course of the two years. The overall mean number of monthly consultations was eight, but the number of queries varied during different months. The lowest number of consultations occurred in November 2021, with only two inquiries, whereas the peak was observed in August 2022, with 15 consultations (Fig. 1C).

## Discussion

This report summarizes the single experience of the ECMM expert consultation service at the Cologne ECMM Excellence Center since August 2021. Most of the overall 189 consultations originated from Germany, while only 14.8% were international. Two thirds of the consultations were related to infections by *Aspergillus* spp., Mucorales, or *Candida* spp. The insights from the data collected provide an understanding of the present challenges faced in the realm of IFI, the importance of a structured consultation system, and the need of international expansion.

The Cologne consultation service proved feasible and with a high level of utilization over a span of two years. While the Cologne ECMM Excellence Center has positioned itself as an international hub for mycological inquiries, it is evident that especially German healthcare professionals in closer proximity to the University Hospital Cologne benefit from this service. The predominantly German origin of consultations underscores the influence of language and proximity on such services. The common language and understanding of the local healthcare context facilitate effective communication and knowledge exchange between the Center and healthcare practitioners, including imaging sharing. In parallel, it implies that additional efforts need to be made for receiving international consultations, in order to place the Cologne ECMM Excellence Center at a global scale. Moreover, while the title of ECMM Excellence Center does not carry an official designation, its acknowledgment can be viewed as an informal nomination, leading to an uptick in consultations from other clinics, both domestically and internationally. Furthermore, the Center's presence and active involvement in national events, initiatives and meetings within Germany may also contribute to the higher number of consultations from this country [29–31]. Closer contact with physicians during these events encourages healthcare professionals to reach out for expert advice and support. More specifically, the data reflects that a majority of consultations originated from North Rhine-Westphalia, Bavaria, and Baden-Württemberg, the top 3 most populated states in the country [32]. This accessibility and collaboration between the Center and healthcare professionals in Germany further enhance the quality of patient care and strengthen the medical mycology community's knowledge-sharing efforts within the country. As mentioned earlier, one of the outstanding responsibilities outlined in the Cologne ECMM Excellence involves addressing a higher volume of international inquiries. To further bolster outreach and engagement, additional promotional activities can be considered. These may include implementing social media campaigns on platforms to share updates and research findings, [33] launching podcast or webinar series featuring experts discussing IFI clinical management. Additionally, interactive workshops and participation in conferences can contribute to a more comprehensive and impactful promotional strategy. Combining these diverse promotional methods can create a robust approach tailored to the goals and target audience of the Cologne ECMM Excellence.

The distribution of pathogens in the consultations showed a pattern that closely resembled the fungal pathogen list compiled by the World Health Organization (WHO) or the local epidemiology of Europe [15, 34–38]. For instance, *Aspergillus* spp. consultations could be potentially linked to the availability of a slightly wider range of treatment options for these pathogens [9, 19], making it a frequent topic for consultation which can be the best approach. Similarly, the higher number of consultations involving *Candida* spp. could be expected, given its status as the most common invasive IFI encountered in clinical settings overall [1]. Additionally, the global concern about multidrug resistance in these two pathogen genera can also be behind, mainly azole-resistant *Aspergillus fumigatus* and *Candida auris*. [39–43] In parallel, the increased awareness of Mucorales infections, [13, 23, 27, 44, 45]. as evidenced by frequent consultations, could be attributed to efforts in educating healthcare professionals about the risks and management of these infections. Moreover, the Cologne ECMM Excellence Center demonstrated its adaptability and proficiency in handling consultations related to less common pathogens, such as phaeohyphomycetes [24, 27]. These consultations highlight the importance of expert advice when dealing with IFI caused by less common species. The drug-related adverse effects and the drug-drug interactions in patients with comorbidities necessitate seeking the best alternative through consultations [46]. Moreover, tailored recommendations based on local capacities and resources can also be provided, serving this as a valuable asset in guiding healthcare professionals in the management of patients with invasive fungal infections in limited resources settings. [13–15, 46, 47]

However, while the Cologne ECMM Excellence Center has been successful in addressing various medical mycology challenges, it also faces certain limitations. One significant limitation is the restricted treatment options available for certain fungal infections. This issue can be particularly challenging, especially when dealing with drug-resistant pathogens or uncommon species for which there may be limited antifungal agents and medical recommendations available [40, 47, 48]. In these situations, the expertise and guidance provided by the Cologne ECMM Excellence Center become invaluable in assisting clinicians in identifying the best possible management strategy. For the future, we would like to enhance the reach and impact of the consultancy platform. To address this, the Center could implement proactive measures, such as targeted marketing, collaborations with medical associations, and partnerships with other organizations, to increase awareness and accessibility of its consultation system among healthcare providers [49]. These efforts can enhance the Center's reach and ensure more healthcare professionals’ benefit from its expertise in managing fungal infections. Ultimately, forthcoming analyses from the ECMM Excellence Centers activity should offer more detailed insights into resolved inquiries. This includes information on the individuals seeking guidance, specifics on the recommendations given (such as alterations in therapeutic approaches or additional diagnostic procedures), and details on patient outcomes. Moreover, it might be beneficial to implement focused initiatives towards continents currently underrepresented in the consultation list, such as Africa or Asia. The limited number of consultations in these regions may be attributed to the absence of traditional cultural or economic ties with Germany. However, in today's globalized environment, where virtually anyone can be reached with a click, we are optimistic that this situation can be effectively addressed and overcome.

In conclusion, the next pivotal steps, applicable to both the Cologne ECMM Excellence Center and its counterparts globally, involve spearheading a systematic and prospective data collection effort across all ECMM Excellence Centers. This entails expanding the dedicated efforts outlined in this manuscript to a global scale. Through active participation in a collaborative, multicenter study, it will be possible to offer a more comprehensive understanding of medical mycology challenges, the impact of expert consultations, and patient outcomes. This proactive and forward-thinking approach holds significant promise for advancing the field of global mycological care, facilitating more informed and evidence-based decision-making in the complex management of IFI.
